# Liver osteopontin is required to prevent the progression of age‐related nonalcoholic fatty liver disease

**DOI:** 10.1111/acel.13183

**Published:** 2020-07-07

**Authors:** Beatriz Gómez‐Santos, Diego Saenz de Urturi, Maitane Nuñez‐García, Francisco Gonzalez‐Romero, Xabier Buque, Igor Aurrekoetxea, Virginia Gutiérrez de Juan, Maria J. Gonzalez‐Rellan, Carmelo García‐Monzón, Águeda González‐Rodríguez, Lorena Mosteiro, Gaizka Errazti, Patricia Mifsut, Sonia Gaztambide, Luis Castaño, Cesar Martin, Rubén Nogueiras, María L. Martinez‐Chantar, Wing‐Kin Syn, Patricia Aspichueta

**Affiliations:** ^1^ Department of Physiology Faculty of Medicine and Nursing University of Basque Country UPV/EHU Leioa Spain; ^2^ Biocruces Bizkaia Health Research Institute Cruces University Hospital Barakaldo Spain; ^3^ Liver Disease Lab, Center for Cooperative Research in Bioscience (CIC bioGUNE), Basque Research and Technology Alliance (BRTA) e Derio Bizkaia Spain; ^4^ Department of Physiology CIMUS University of Santiago de Compostela‐Instituto de Investigación Sanitaria Santiago de Compostela Spain; ^5^ CIBER Fisiopatología de la Obesidad y Nutrición (CIBERobn) Madrid Spain; ^6^ Liver Research Unit Santa Cristina University Hospital Instituto de Investigación Sanitaria Princesa Madrid Spain; ^7^ Centro de investigación Biomédica en Red de Enfermedades Hepáticas y Digestivas (CIBERehd), Carlos III National Health Institute Madrid Spain; ^8^ Department of Biochemistry and Molecular Biology Biofisika Institute (UPV/EHU, CSIC) UPV/EHU Spain; ^9^ Section of Gastroenterology Ralph H Johnson VAMC Charleston SC USA; ^10^ Division of Gastroenterology and Hepatology Medical University of South Carolina Charleston SC USA

**Keywords:** aging, lipid metabolism, nonalcoholic fatty liver disease, Osteopontin, p53, senescence

## Abstract

Osteopontin (OPN), a senescence‐associated secretory phenotype factor, is increased in patients with nonalcoholic fatty liver disease (NAFLD). Cellular senescence has been associated with age‐dependent hepatosteatosis. Thus, we investigated the role of OPN in the age‐related hepatosteatosis. For this, human serum samples, animal models of aging, and cell lines in which senescence was induced were used. Metabolic fluxes, lipid, and protein concentration were determined. Among individuals with a normal liver, we observed a positive correlation between serum OPN levels and increasing age. This correlation with age, however, was absent in patients with NAFLD. In wild‐type (WT) mice, serum and liver OPN were increased at 10 months old (m) along with liver p53 levels and remained elevated at 20m. Markers of liver senescence increased in association with synthesis and concentration of triglycerides (TG) in 10m OPN‐deficient (KO) hepatocytes when compared to WT hepatocytes. These changes in senescence and lipid metabolism in 10m OPN‐KO mice liver were associated with the decrease of 78 kDa glucose‐regulated protein (GRP78), induction of ER stress, and the increase in fatty acid synthase and CD36 levels. OPN deficiency in senescent cells also diminished GRP78, the accumulation of intracellular TG, and the increase in CD36 levels. In 20m mice, OPN loss led to increased liver fibrosis. Finally, we showed that OPN expression in vitro and in vivo was regulated by p53. In conclusion, OPN deficiency leads to earlier cellular senescence, ER stress, and TG accumulation during aging. The p53‐OPN axis is required to inhibit the onset of age‐related hepatosteatosis.

## Graphical abstract

OPN is a protective factor required to preserve liver health during aging. As OPN‐deficient mice become older, increased levels of senescence, ER stress, hepatosteatosis, DNA damage, fibrosis, and inflammation appear.

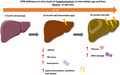

## 1 INTRODUCTION

Nonalcoholic fatty liver disease (NAFLD) is one of the most common causes of liver disease in Western countries and includes a spectrum of disorders (Friedman, Neuschwander‐Tetri, Rinella, & Sanyal, 2018). Hepatosteatosis is the earliest stage, and it is caused by the accumulation of fat in the hepatocytes. Increased fatty acid (FA) uptake from the diet and/or peripheral tissues, increased lipogenesis, defects in lipid oxidation, and/or export to circulation in VLDL particles induce their aberrant accumulation that can lead, in some patients, to progression to nonalcoholic steatohepatitis (NASH), fibrosis, cirrhosis, and/or hepatocellular carcinoma (HCC) (Friedman et al., 2018).

Epidemiological studies have demonstrated that NAFLD and NASH are common among the elderly (Bertolotti et al., 2014). In old age, fat is redistributed outside the usual fat deposits and lipids can accumulate in nonadipose tissues like skeletal muscle, heart, and liver. Thus, aging is associated with an increase in the lipid accumulation within the liver that may compromise the normal function due to lipotoxicity (Slawik & Vidal‐Puig, 2006).

Osteopontin (OPN) is a multifunctional protein that is expressed in a variety of tissues and has many functions, both physiological and pathological (Ashkar et al., 2000; Ramaiah & Rittling, 2007). It is involved in liver pathologies and acts as a modulator of liver lipid metabolism (Nunez‐Garcia et al., 2017; Nuñez‐Garcia et al., 2018). OPN is involved in NAFLD pathogenesis (Kiefer et al., 2011), and its liver expression is increased in obesity and correlates with steatosis and insulin resistance (Gómez‐Ambrosi et al., 2007). OPN expression is also upregulated in liver cancers such as in HCC and cholangiocarcinoma (Wen, Jeong, Xia, & Kong, 2016; Zheng et al., 2018). Regarding its role as a metabolic modulator, it controls the fate of acetyl‐CoA in liver (Nunez‐Garcia et al., 2017; Nuñez‐Garcia et al., 2018) and rewires liver lipid metabolism after partial hepatectomy (Nuñez‐Garcia et al., 2018). OPN is considered a senescence‐associated secretory phenotype (SASP) factor (Flanagan et al., 2017; Pazolli et al., 2009). SASP factors are mainly cytokines, chemokines, and proteases with autocrine and paracrine effects that affect the neighboring cells and are able to induce senescence (Coppé, Desprez, Krtolica, & Campisi, 2010; Malaquin, Martinez, & Rodier, 2016). The accumulation of senescent cells can lead to inflammation and in turn lead to further senescence of surrounding cells. SASP may be beneficial because it can help maintain homeostasis by clearing senescent cells and, thereby, reduce the tissue damage; however, if senescent cells are not cleared and accumulated, detrimental effects appear contributing to inflammation and to making surrounding cells senescent (Lau & David, 2019; Malaquin et al., 2016).

Senescence is a stable cell cycle arrest mediated through activation of p53/p21 and/or Rb/p16 pathways (Munoz‐Espin & Serrano, 2014). Senescent cells have been found in the livers of NAFLD and cirrhotic patients and of high‐fat diet fed and genetically obese mice (Aravinthan & Alexander, 2016; Ogrodnik et al., 2017). Cellular senescence also drives age‐dependent hepatosteatosis (Ogrodnik et al., 2017). However, the mechanisms involved in senescence‐induced NAFLD progression remain poorly understood. It is recognized that the aging liver is potentially at risk of injury because of its inability to respond to stresses. In the aging liver, a significant proportion of hepatocytes develop a senescent phenotype (Wang et al., 2009), and the aging liver exhibits decreased capacity for liver regeneration (Schmucker & Sanchez, 2011) and is less able to cope with oxidative stress (Schmucker, 2005). The aging liver also expresses lower levels of chaperone proteins (Erickson, Dunning, & Holtzman, 2006) and exhibits increased levels of cellular apoptosis (Zhong et al., 2017).

Here, we investigated the role of OPN in the aging‐related metabolic fatty liver disease. We used serum samples from obese and nonobese patients, animal models of aging, and cell lines and found that OPN is necessary to prevent age‐related liver disease. The results showed that the loss of OPN leads to earlier cellular senescence, ER stress, increased *de novo* lipogenesis, and increased lipid uptake, altogether promoting fatty liver disease. This study also demonstrated that expression of liver OPN is regulated, at least in part, by p53 in cellular models of senescence and NAFLD.

## 2 RESULTS

### 2.1 Aging increases osteopontin in liver and serum

**FIGURE 1:**
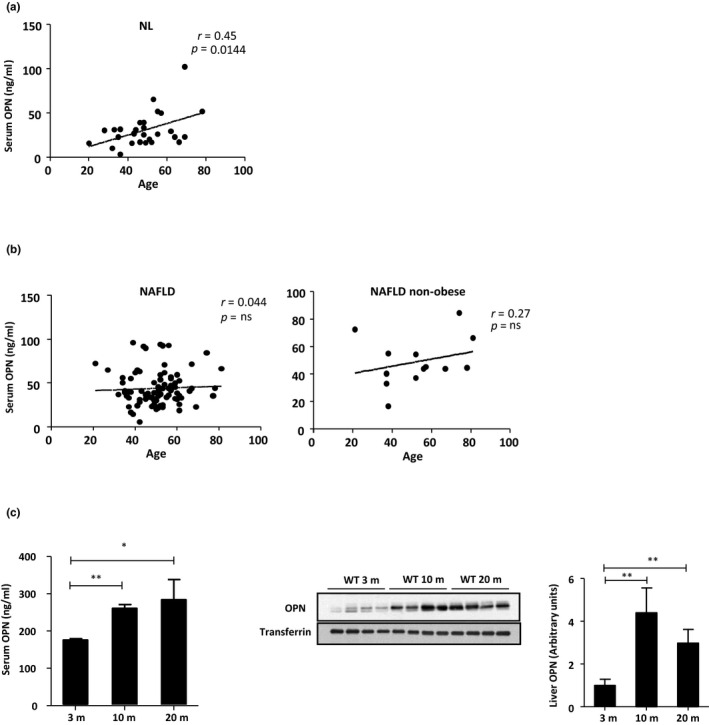
Osteopontin serum and liver levels increase during aging. (a) Serum samples from normal liver (NL) individuals (*n* = 34), (b) nonalcoholic fatty liver disease (NAFLD) patients (*n* = 89), and nonobese NAFLD patients (*n* = 13) were quantified by ELISA to asses circulating OPN levels. (c) Serum OPN from 3‐, 10‐, and 20‐month‐old (m) wild‐type (WT) mice was quantified by ELISA and liver OPN levels were assessed by immunoblotting using transferrin as loading control (*n* = 4–8). Correlation analysis was tested using the Pearson correlation test. Values are means ± *SEM*. Significant differences are denoted by *p < 0.05, **p < 0.01, and ***p < 0.001 (Student's *t* test)

As OPN is secreted into the bloodstream, we first assessed if OPN is associated with aging by measuring serum OPN levels in a cohort of individuals of varying ages and who were found to have a normal liver (NL) (*n* = 34) (Figure 1a) (Table 1). The results showed a positive correlation between serum OPN concentration and age (Figure 1a). Given that OPN is increased in liver (Lima‐Cabello et al., 2010) and serum of NAFLD patients (Nunez‐Garcia et al., 2017), OPN levels were also measured in a cohort of NAFLD patients (*n* = 89) (Figure 1b) (Table 1). The correlation between serum OPN concentration and age was lost in NAFLD patients, in which OPN levels were already high in younger patients (Figure 1b).

**TABLE 1:** Demographic, metabolic, biochemical, and histological characteristics of individuals with normal liver (NL), nonalcoholic fatty liver disease (NAFLD), and nonobese NAFLD. Obese patients were considered when the BMI was ≥30

	NL (*n* = 34)	NAFLD (*n* = 89)	nonobese NAFLD (*n* = 13)
BMI (kg/m^2^)	28.1	39.42***	25.96***
Age(Years)	48.3	50.5	52.92
Male gender %	32.4	31.7	46
Female gender %	67.6	68.3	54
TG (mg/dl)	104.3	144.51***	128.1*
Glucose (mg/dl)	95.4	93.7	104
CHOL (mg/dl)	188.4	169.41*	201.15***
CHOL‐HDL (mg/dl)	49.9	38.86***	53.15***
ALT (IU/L)	19.2	40.68***	34.620
AST (IU/L)	18.1	29.55***	25.690
Insulin (mU/ml)	6.6	11.76***	6.9
Steatosis %			
Grade 0	100	0	0
Grade 1		58.0	84.6
Grade 2		27.0	15.4
Grade 3		15.0	0

Note

Data are the mean or percentage (%). Values are means of *n* = 34 for NL, *n* = 89 for NAFLD and *n* = 13 for nonobese NAFLD patients. Significant differences are denoted by *p < 0.05, **p < 0.01, and ***p < 0.001 when comparing NL versus NAFLD, and when comparing NAFLD versus nonobese NAFLD (Student's *t* test).

Abbreviations: ALT, alanine aminotransferase; AST, aspartate aminotransferase; BMI, body mass index; CHOL, cholesterol; HDL, high‐density lipoprotein; TG, triglyceride.

To investigate the involvement of OPN in age‐related development of fatty liver, animal models that recapitulate aging were generated. In this model, 3‐month‐old (3m) mice represent young mice, 10‐month‐old (10m) mice intermediate‐age mice, and 20‐month‐old mice (20m) aged mice. The results showed that serum OPN concentration increased from 3 to 10m, and the increase maintained in 20m mice (Figure 1c). Total Liver OPN levels were also analyzed, and a similar profile was obtained. OPN protein levels were increased in 10m mice as compared to 3m mice, and these levels were maintained at 20m (Figure 1c).

### 2.2 OPN deficiency in mice results in an early increase in liver lipid storage during aging

Given that OPN levels increase in liver during aging, we evaluated the role of OPN in liver lipid accumulation and NAFLD development. For this, we used 3m, 10m and 20m WT and OPN‐knockout (KO) mice. To study the role of OPN in NAFLD and aging, a separate group of WT and OPN‐KO mice were fed a high‐fat diet (HFD) from 16m until sacrifice at 20m (i.e., mice were 4 months on HFD).

**FIGURE 2:**
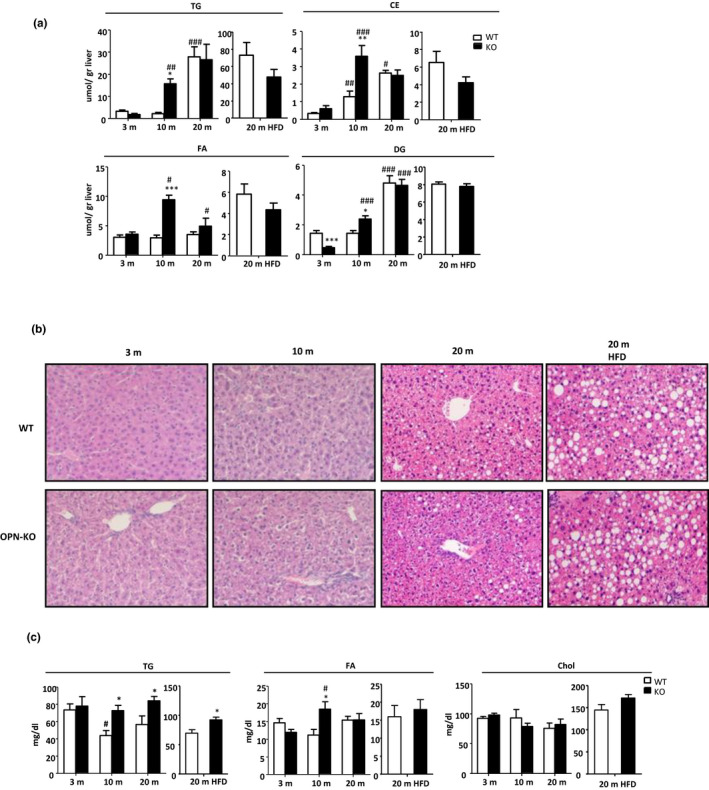
OPN deficiency leads to a premature increase of liver lipid accumulation. (a) Lipids were extracted from liver homogenates of (*n* = 4–8) 3‐, 10‐, and 20‐month‐old (m) WT and OPN‐KO mice fed a chow diet (CD) and a high‐fat diet (HFD). Triglycerides (TG), cholesteryl esters (CE), diglycerides (DG), and fatty acids (FA) were separated and quantified. (b) H&E staining was performed to study the histology of the liver. (c) Serum lipids TG, FAs, and total cholesterol (Cho) were quantified (*n* = 4–8) in 3, 10, and 20 m WT and OPN‐KO mice fed a CD and a HFD. Values are means ± *SEM*. Significant differences when comparing genotypes of the same age‐group are denoted by *p < 0.05, **p < 0.01, and ***p < 0.001 and #*p* < 0.05, ##*p* < 0.01, and ###*p* < 0.001 when comparing with previous age‐group in the same genotype (Student's *t* test)

Liver lipid analysis showed a premature increase in lipid concentration in 10m OPN‐KO mice compared to their age‐matched WTs (Figure 2a). In fact, there was a rise in triglycerides (TG), cholesteryl ester (CE), fatty acids (FA), and diglycerides (DG) concentration at 10m that maintained at 20m. Thus, during aging, fluctuations in liver lipid concentration differ between WT and OPN‐KO mice (Figure 2a); in WT mice, the peak of concentration for all lipids was observed at 20m, being lipid storage similar at 3 and 10m (Figure 2a,b); OPN‐KO mice on the other hand accumulated liver lipids at an earlier age. Even more, the results showed that serum TG was increased in 10m and 20m OPN‐KO mice when compared to their WT controls (Figure 2c). Serum FAs also increased in 10m OPN‐KO animals. However, serum total cholesterol (Chol) did not change when comparing age or genotype (Figure 2c).

Others have previously reported that young OPN‐KO mice are protected from diet‐induced hepatosteatosis (Kiefer et al., 2011; Lancha et al., 2014). However, given the observed early accumulation of lipid in the OPN‐KO mice liver during aging, lipid storage and hepatosteatosis were also measured in the HFD‐fed 20m mice. The lipid concentration (Figure 2a) and accumulation of lipid droplets (Figure 2b) showed that aged OPN‐KO mice were no longer protected from diet‐induced hepatosteatosis. The results showed that at 20m, the *de novo* lipogenesis was decreased (Figure S1A); however, increased esterification of fatty acids into more complex lipids (Figure S1B) associated with increased levels of the fatty acid transporter CD36 were observed (Figure S1C). Besides, serum TG levels were increased (Figure 2c). Although the final body weight of HFD‐fed 20m OPN‐KO mice was similar to their age‐matched HFD‐fed WT mice (Figure S1D), they developed a greater degree of insulin resistance as demonstrated by the insulin tolerance test (ITT) (Figure S1E), insulin levels, and HOMA‐IR (Figure S1F).

### 2.3 OPN deficiency induces senescence‐associated hepatosteatosis

**FIGURE 3:**
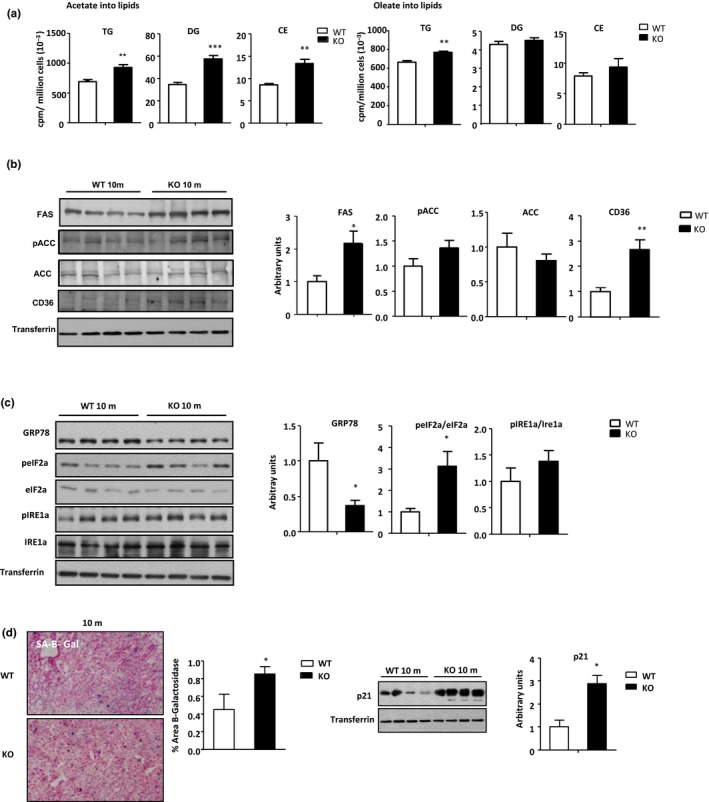
Increased lipid synthesis is linked to markers of senescence and ER stress in 10‐month‐old OPN‐KO mice liver. (a) Hepatocytes isolated from 10‐month‐old (m) WT and OPN‐KO mice were cultured in medium with [^3^H]acetate or [^3^H]oleate for 4 hr. Lipids were extracted and radioactivity incorporated into TG, DG, and CE was assessed by liquid scintillation (*n* = 5). (b) Fatty acid synthase (FAS), phosphorylated, and total acetyl‐CoA carboxylase (ACC) and CD36 protein levels of 10 m WT and OPN‐KO mice were assessed by immunoblotting using transferrin as loading control (*n* = 5–8). (c) Immunoblot analysis of 10 m mice of GRP78, total and phosphorylated eIF2α and IRe1a protein from liver extract was assessed using transferrin as loading control (*n* = 5–6). (d) Liver senescence‐associated (SA) β‐galactosidase‐positive area percentage was quantified in 10 m WT and OPN‐KO mice. Immunoblot analysis of p21 protein from liver extract was assessed using transferrin as loading control (*n* = 5). Significant differences when comparing genotypes of the same age‐group are denoted by *p < 0.05, **p < 0.01, and ***p < 0.001 (Student's *t* test)

Increased lipid storage in liver is due to a misbalance between processes that control lipid input and output. To ascertain the mechanisms involved in the increased lipid storage, *de novo* lipogenesis and the esterification of fatty acids were measured. We used hepatocytes from 10m WT and OPN‐KO mice because 10m OPN‐KO mice exhibited upregulation in liver and serum lipids. The results showed that *de novo* synthesis of TG, DG, and CE and the esterification of oleate into TGs were increased in OPN‐KO mice (Figure 3a), but no changes were observed in the incorporation of oleate into DG or CE (Figure 3a). Protein levels of fatty acid synthase (FAS) which is involved in fatty acid synthesis, and CD36 which is involved in fatty acid uptake (Figure 3b), were also augmented, but no significant changes were observed in the pACC and ACC levels (Figure 3b). Catabolism of fatty acids through beta oxidation (β‐oxidation) (a mechanism involved in lipid output) was also assessed by the production of acid soluble metabolites (ASM) and CO_2_. We did not detect any statistically significant difference in palmitate β‐oxidation between both groups of mice (Figure S2A).

Loss of proteostasis is a hallmark of aging (López‐Otín, Blasco, Partridge, Serrano, & Kroemer, 2013). The endoplasmic reticulum (ER) is an important organelle for regulating lipid homeostasis and protein synthesis (Han & Kaufman, 2016). Many insults can disturb ER homeostasis, which lead to ER stress and activation of the unfolded protein response (UPR). The UPR might lead to increased lipogenesis, thus contributing to lipotoxicity. On the other hand, lipid accumulation *per se* can also perturb ER function, which then generates ER stress (Basseri & Austin, 2012). ER stress, therefore, can activate pathways involved in accumulation of lipids in hepatocytes, which in turn exacerbates ER stress‐mediated increase in lipid storage and induce a vicious positive‐feedback cycle, and persistence of ER stress may lead to development and/or progression of liver disease (Lebeaupin et al., 2018).

The decrease in the amount of chaperone proteins is a hallmark of aging (Erickson et al., 2006) and may be an activator of ER stress (Klaips, Jayaraj, & Hartl, 2018). Thus, we next investigated whether the early lipid accumulation in OPN‐KO mice during aging could be associated with altered levels of chaperones. Interestingly, the results showed that in 10m OPN‐KO mice livers, levels of the chaperone GRP78 were decreased while levels of phosphorylated eIF2α (peIF2α) and the peIF2α/eIF2α ratio were increased (Figure 3c). No changes were observed in the IRE1α branch of UPR (Figure 3c). Altered mTOR signaling could be linked to the activation of ER stress (Liu & Sabatini, 2020), the results showed that phosphorylation of mTOR was decreased in OPN‐KO mice (Figure S2B), while there was a tendency toward decrease in that of S6 (Figure S2B).

It is also well recognized that proteostasis prevents cellular senescence (Kim et al., 2018), and senescent cells can contribute to age‐related tissue degeneration and fat accumulation (Ogrodnik et al., 2017). Therefore, we also analyzed markers of senescence in 10m mice livers. In 10m OPN‐KO mice livers, the increased β‐galactosidase (SA‐β‐galactosidase) and p21 levels (Figure 3d) showed the induction of a senescence phenotype in liver when compared to the age‐matched WT mice. Levels of γH2AX, indicating DNA damage, were also measured; however, they were undetectable (data not shown).

Considering that deficient autophagy in the liver impairs the mobilization of TGs into FAs, resulting in steatosis (Singh et al., 2009) and that it could have a direct effect on triggering senescence and quiescence (Rajendran et al., 2019), we analyzed levels of proteins involved in autophagy. We found no statistically significant differences in ATG5, ATG7, or LC3 levels in 10m OPN‐KO mice (Figure S2C).

**FIGURE 4:**
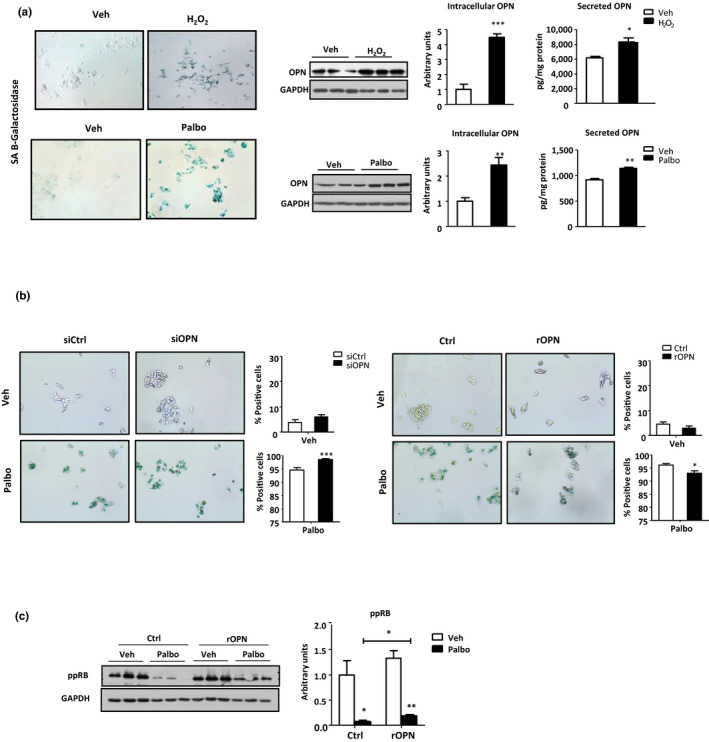
OPN deficient cells are more vulnerable to senescence. (a) HepG2 cells were treated with hydrogen peroxide (H_2_O_2_), Palbociclib (Palbo) (2 µM), or vehicle (Veh). Senescence induction was assessed with the senescence‐associated (SA)‐β‐galactosidase staining. OPN protein levels from HepG2 cells were measured by immunoblotting using glyceraldehyde‐3‐phosphate dehydrogenase (GAPDH) as loading control. Secreted OPN in the media was measured by ELISA (*n* = 4–7). (b) OPN‐deficient cells (siOPN), their controls (siCtrl), control (Ctrl), and recombinant OPN (rOPN) treated HepG2 cells were incubated either with Palbo (2 µM) to induce senescence or with Veh. SA‐β‐galactosidase staining was performed, and the percentage of positive cells was evaluated (*n* = 4–6). (c) rOPN‐treated cells (rOPN) and their control cells (Ctrol) were treated with Palbo (2 µM), to induce senescence, or vehicle (Veh). Phosphorylated Rb protein (ppRB) levels were measured by immunoblotting to study palbociclib effect using GAPDH as loading control (*n* = 3). Values are means ± *SEM*. Significant differences are denoted by *p < 0.05, **p < 0.01, and ***p < 0.001 (Student's *t* test)

To establish a causality of events, and to validate these results in an *in vitro* model of senescence, palbociclib (Palbo) (a cyclin‐dependent protein kinase 4/6 inhibitor) or hydrogen peroxide (H_2_O_2_) were used to induce senescence in HepG2 cells. As OPN is a SASP factor and senescence is a hallmark of aging, intracellular and secreted OPN levels were measured in HepG2 cells after the induction of senescence. Induction of senescence, as the SA‐β‐galactosidase staining showed (Figure 4a), increased intracellular and secreted OPN protein levels (Figure 4a). When OPN knocked‐down (siOPN) (Figure S3A) cells where treated with vehicle no differences were observed in SA‐β‐galactosidase‐positive cells as compared to their controls (Figure 4b). However, when cells were treated with palbo, siOPN cells showed higher percentage of SA‐β‐galactosidase‐positive cells as compared to the siCtrl cells (Figure 4b). Conversely, when cells were treated with rOPN, there was a significant decrease of SA‐β‐galactosidase‐positive cells (Figure 4b), and a reduction of RB phosphorylation (target of CDK4/6) (Figure 4c), thus confirming the role of OPN as a factor that prevents senescence.

**FIGURE 5:**
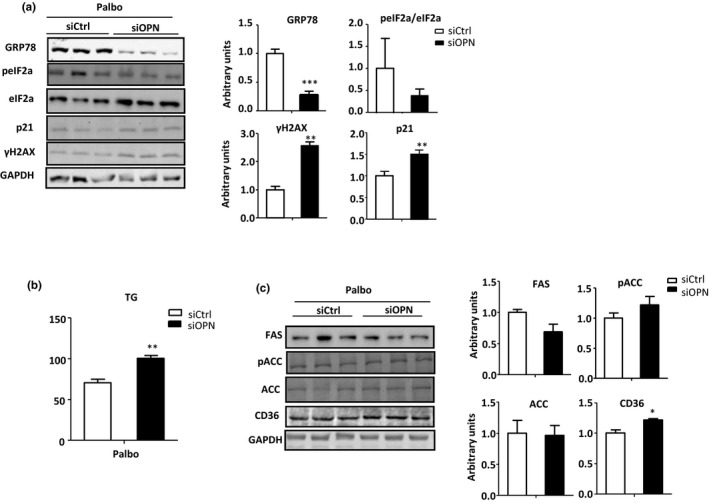
Lack of OPN in senescent cells induces the decrease of GRP78 and the storage of triglycerides. OPN‐deficient cells (siOPN) and their controls (siCtrl) were treated with Palbociclib (Palbo) to induce senescence. (a) GRP78, total and phosphorylated eIF2α, p21, and γH2AX protein levels were measured by immunoblotting using glyceraldehyde‐3‐phosphate dehydrogenase (GAPDH) as loading control (*n* = 4–8). (b) Intracellular TG levels were measured (C) FAS, total and phosphorylated acetyl‐CoA carboxylase (ACC), and CD36 protein levels were measured using GAPDH as a loading control (*n* = 4). Values are means ± *SEM*. Significant differences are denoted by *p < 0.05, **p < 0.01, and ***p < 0.001 (Student's *t* test)

OPN knockdown (i.e., siOPN) during palbo‐associated senescence also led to decreased GRP78 but increased γH2AX and p21 level expression (Figure 5a). By contrast, GRP78 levels remained unaltered and γH2AX decreased, while p21 levels increased (Figure S3B) when OPN knockdown occurred under normal (i.e., non senescence) conditions. In sum, these results indicate that 1‐the induction of senescent cells *in vitro* promotes the generation and secretion of OPN; and 2‐the induction of senescence *in vitro* is more marked when OPN is absent.

Changes in levels of GRP78 and markers of senescence during palbo treatment were also associated with an increase in HepG2 TG concentration (Figure 5b) and the upregulation of CD36 (Figure 5c). No changes, however, were observed in levels of the pro‐lipogenic proteins, FAS or ACC (Figure 5c and Figure S3D). It was recently reported that CD36 is rapidly upregulated in multiple cell types in response to senescent stimuli (Chong et al., 2018). By comparison, TG and CD36 levels maintained unchanged when HepG2 cells were not treated with the senescence inducer (Figure S3C,D). Thus, OPN deficiency increases cellular vulnerability to senescence and the senescent‐associated altered processes. These in vitro experiments recapitulate results obtained in vivo, and imply that reduced OPN levels in the setting of cellular senescence will increase lipid storage in vivo, will enhance activation of ER stress and de novo lipogenesis.

### 2.4 OPN deficiency promotes age‐related fibrosis

**FIGURE 6:**
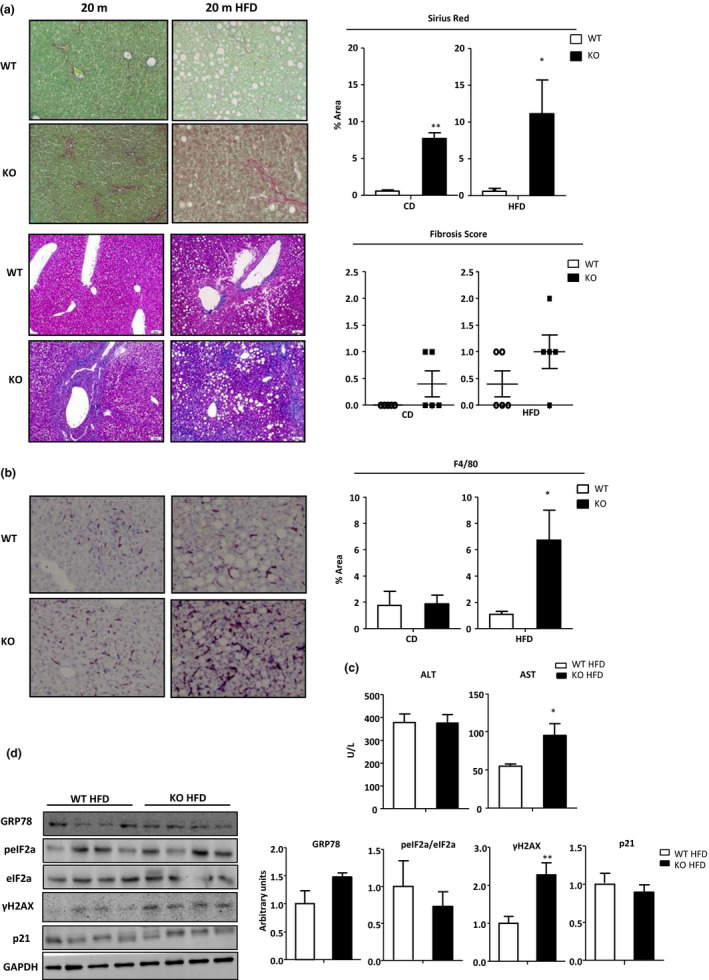
Liver fibrosis is increased in aged high‐fat diet fed OPN‐KO mice. (a) Sirius Red and Masson trichrome stainings were performed and evaluated to assess tissue fibrosis in 20‐month‐old (m) WT and OPN‐KO mice fed a chow (CD) and a high‐fat diet (HFD) (*n* = 4–6). (b) F4/80 staining was performed in 20 m mice fed a CD and a HFD for evaluating inflammation. (c) ALT and AST serum levels were evaluated in 20 m mice fed a HFD (*n* = 4–5). (d) Immunoblot analysis of GRP78, total and phosphorylated eIF2a, γH2Ax, and p21 protein levels from liver extract were assessed using transferrin as loading control (*n* = 4–5) in 20 m HFD‐fed WT and OPN‐KO mice. Values are means ± *SEM*. Significant differences are denoted by *p < 0.05, **p < 0.01, and ***p < 0.001 when comparing WT and OPN‐KO mice (Student's *t* test)

Human NASH and fibrosis are related to increased OPN levels (Glass et al., 2018), and the current results show that liver OPN prevents age‐related early accumulation of lipids, senescence, and ER stress. We next evaluated whether OPN was also required to prevent fibrosis in the aged mice. The results showed that at 3m and 10m, fibrosis in OPN‐KO mice was comparable with age‐matched WT mice (Figure S4A). Similar results were noted for F4/80, a marker of macrophages/ kupffer cells (Figure S4A), and SA‐β‐galactosidase (Figure S2D). 20m OPN‐KO mice however developed significantly more liver fibrosis (Figure 6a) and had higher levels of F4/80 protein (Figure 6b) compared with WT mice under HFD conditions. AST levels were also significantly higher in 20m HFD‐fed OPN‐KO mice (Figure 6c). Fibrosis was also upregulated in the CD‐fed 20m OPN‐KO mice while F4/80 immunostaining remained unaltered (Figure 6a,b). Even though fibrosis and F4/80 increased in the HFD‐fed 20m OPN‐KO mice, lipid storage maintained unaltered (Figure 2a). We next analyzed induction of ER stress and senescence. The results showed that while GRP78, activation of the PERK branch of UPR and p21 levels remained unaltered in HFD‐fed 20m OPN‐KO mice livers as compared to their HFD‐fed 20m WT mice (Figure 6d), levels of γH2AX, indicating DNA damage, were increased as compared to their controls (Figure 6d). The latter was not observed when mice were fed a chow diet (Figure S4B).

### 2.5 Liver OPN is p53 regulated

**FIGURE 7:**
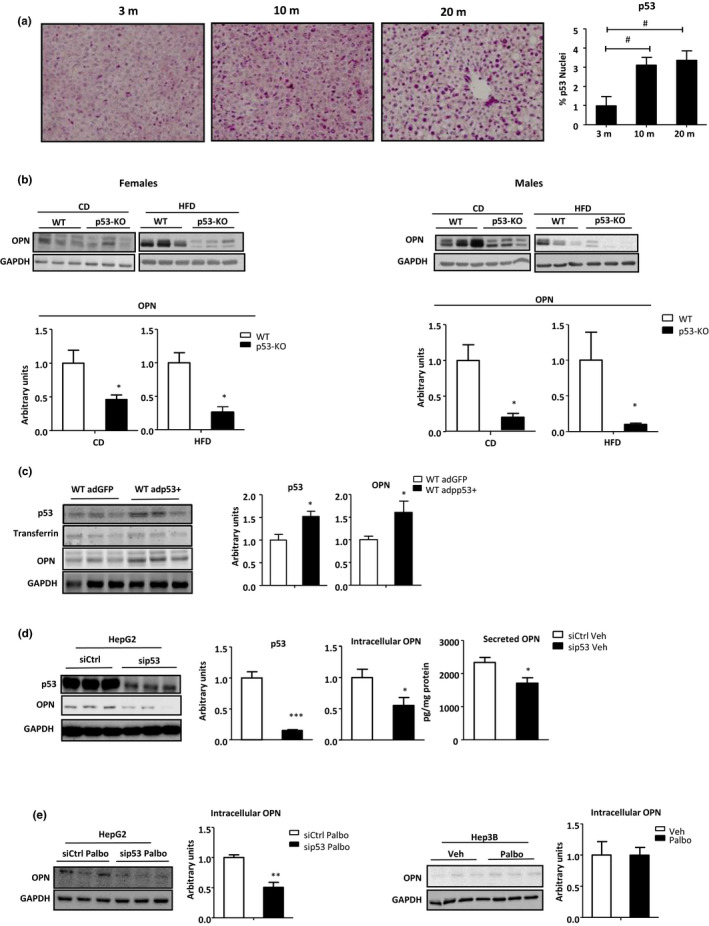
Liver OPN is p53 regulated. (a) Protein levels of p53 were evaluated by immunohistochemistry in liver sections of 3‐, 10‐, and 20‐month‐old (m) wild‐type (WT) mice (*n* = 3–5). (b) OPN protein levels from liver homogenates were measured in WT and p53‐KO male and female mice fed a chow diet (CD) and a high‐fat diet (HFD) by immunoblotting using glyceraldehyde‐3‐phosphate dehydrogenase (GAPDH) as loading control (*n* = 4–6). (c) Liver p53 and OPN protein levels were evaluated in WT mice fed a HFD injected with p53‐dominant positive adenovirus (adp53) and GFP (adGFP), using transferrin or GAPDH as a loading control (*n* = 4–6). (d) Protein levels of p53 and OPN in siCtrl and sip53 HepG2 cells were measured by immunoblotting using GAPDH as loading control. Extracellular OPN was measured using an ELISA (*n* = 4–5). (e) OPN protein levels from HepG2 cells silenced for p53 and Hep3B cells treated with palbociclib (Palbo) were measured by immunoblotting using GAPDH as loading control. OPN media levels from Hep3B cells treated either with vehicle (Veh) or with palbociclib (Palbo) were measured by ELISA (*n* = 4–8). Values are means ± *SEM*. Significant differences are denoted by *p < 0.05, **p < 0.01, and ***p < 0.001 (Student's *t* test)

p53 signaling mediates cellular senescence, and it is involved in aging (Munoz‐Espin & Serrano, 2014; Rufini, Tucci, Celardo, & Melino, 2013). p53 and other p53 family members also regulate metabolic pathways (Napoli & Flores, 2017; Porteiro, Fondevila, Delgado, et al., 2017) and the development of hepatosteatosis (Porteiro, Fondevila, Buque, et al., 2017). OPN has been described as a p53 target in fibroblasts (Morimoto, Sasaki, Ishida, Imai, & Tokino, 2002). As both OPN and p53 play a role in senescence, lipid metabolism and hepatosteatosis, we analyzed if OPN could also be a target of p53 in liver. Interestingly, the data showed that liver p53 levels increased during aging, mirroring a similar pattern to OPN in the animal model of aging (Figure 7a), and that loss of p53 resulted in a dramatic decrease in OPN in both CD‐ and HFD‐fed mice; the decrease was particularly marked among HFD‐fed mice (Figure 7b). These data are consistent with prior reports, which showed that liver OPN increased with HFD (Bertola et al., 2009). Over‐expression of p53 in vivo, as others performed before (Porteiro, Fondevila, Delgado, et al., 2017), also increased liver OPN levels (Figure 7c), even in p53‐KO mice (Figure S5A).

Data obtained from the Cell Line Encyclopedia (© 2019 The Broad Institute of MIT & Harvard) showed that the cell line with the least p53 expression, the Hep3B, also had the lowest OPN expression. *In vitro* experiments showed that the decreased p53 levels in HepG2 cells using a siRNA led to diminish intracellular and secreted OPN levels (Figure 7d). Earlier, we showed that induction of senescence with Palbo increased OPN levels (Figure 4a). Here, we found that cells deficient in p53 (i.e., either p53‐silenced cells or p53‐null cells) were unable to upregulate OPN expression even after treatment with Palbo (Figure 7e; Figure S5B) or H_2_O_2_ (Figure S5C), confirming that p53 is a master regulator of hepatocyte OPN expression.

## 3 DISCUSSION

Aging is a complex multifunctional process, in which metabolism plays an important role (López‐Otín et al., 2013). Indeed, many aging‐related diseases have a metabolic component. Epidemiological studies have demonstrated that NAFLD and NASH are common among the elderly (Bertolotti et al., 2014) although some pieces of evidence suggest that very old age‐groups show a decrease in NAFLD and HCC prevalence (Sheedfar, Biase, Koonen, & Vinciguerra, 2013). Senescence is one of the hallmarks of aging and is a permanent state of cell cycle arrest in response to different stresses, thus, a cellular defense mechanism (López‐Otín et al., 2013). This process takes place in several tissues during different physiological and pathological conditions. Senescence of hepatocytes is a feature of chronic liver disease independent of etiology and plays an important role in the progression of chronic liver disease (Aravinthan & Alexander, 2016). Cellular senescence has been associated with age‐dependent hepatosteatosis, and it correlates with severity of NAFLD (Ogrodnik et al., 2017). Specifically, the accumulation of senescent hepatocytes has been shown to promote progression (Aravinthan & Alexander, 2016). Previous work has described osteopontin (OPN) as a senescence‐associated secretory phenotype (SASP) factor (Flanagan et al., 2017; Pazolli et al., 2009). Although senescence‐associated cell cycle exit likely evolved as an antitumor mechanism, SASP contains both anti‐ and pro‐tumorigenic potential (Lau & David, 2019). Thus, identification and characterization of the SASP factors that are pro‐ and those that are antitumorigenic depending on the contexts and tissue is crucial. Here, we investigated the role of OPN in the age‐related hepatosteatosis. Data showed a positive correlation between serum OPN levels and increasing age in humans. Correlation however, lost in NAFLD patients where OPN levels are already higher in younger patients. Similar results were obtained in mice, where liver and serum OPN increases with age, mirroring the same pattern as p53, a regulator of cellular senescence, and whose downregulation has been linked to hepatosteatosis and induction of ER stress (Porteiro, Fondevila, Delgado, et al., 2017). Finally, we also found that when senescence was induced in HepG2 cells, OPN levels were also increased in cells and culture medium.

Thus, to determine whether the age‐related increase in OPN was protective or deleterious for hepatosteatosis and liver disease progression; lipid storage, metabolic fluxes, ER stress, and senescence were also studied in OPN‐KO mice during aging. Young OPN‐KO mice are protected from diet‐induced hepatosteatosis (Kiefer et al., 2011; Lancha et al., 2014). Older mice, which lacked OPN however, accumulated liver lipids, developed liver steatosis, and had higher serum TG levels in association with insulin resistance at an earlier age than the WT mice.

We previously observed that in young mice, OPN is a regulator of liver lipid metabolism during regeneration after partial hepatectomy (Nuñez‐Garcia et al., 2018). As intermediate‐age (10m) OPN‐KO mice was noted to store more lipids, we investigated the mechanisms by which OPN regulate liver lipid metabolism. We found that increased lipid synthesis and uptake were linked to higher levels of fatty acid synthase (FAS) and CD36. We also found that markers of senescence and activation of the unfolded protein response PERK branch (linked to decreased GRP78 chaperone levels) were increased. Loss of proteostasis and senescence are hallmarks of aging (López‐Otín et al., 2013), and several studies have demonstrated that the decrease in chaperone number is associated with aging (Lee et al., 2017) and can be a cause of the increased ER stress with aging. Other studies have also shown that forced over‐expression of GRP78 attenuates steatosis by inhibiting sterol regulatory element‐binding protein (SREBP‐1c) (Kammoun et al., 2009). Thus, the decrease in GRP78 levels may activate SREBP‐1c (given that FAS is increased and is one of its downstream targets) and induce *de novo* lipid synthesis.

OPN has been linked to fibrosis and NASH in humans and animal models. In fact, OPN has been described as a profibrogenic factor and its deficiency avoids fibrosis (Coombes et al., 2015). The role of OPN however is quite different during aging. Liver fibrosis was increased in 20m (aged) OPN‐KO mice, and this was enhanced after a HFD. Induction of fibrosis by senescent hepatocytes is generally a way of limiting tissue injury as part of wound healing process. The accumulation of senescent hepatocytes leads to continued activation of hepatic stellate cells, which in turn, leads to liver fibrosis (Aravinthan & Alexander, 2016). Liver fibrosis (observed mainly in HFD‐fed OPN‐KO mice) was associated with inflammation, DNA damage, and insulin resistance. In combination, these factors contribute to the development of liver fibrosis. It is unclear however whether the inflammation and fibrosis observed in the aging mice are consequences of a premature liver lipid storage or are dependent on other dysregulated mechanisms that activate during aging. Supporting the protective role of OPN, other studies have also shown a protective role of OPN in alcoholic hepatitis (Lazaro et al., 2015; Magdaleno et al., 2018).

Finally, previous work has shown that OPN is a p53 target in fibroblasts (Morimoto et al., 2002). p53 represses some of the SASP in certain conditions and cell types (Wiley et al., 2018). However, p53 also plays a critical enhancing role in SASP production, as many of these SASP factors are directly stimulated by p53, among them the secretion of several cytokines (Pavlakis & Stiewe, 2020). SASP profile can greatly variate depending on the cell type and effector. Concerning liver disease, it has been demonstrated that p53 regulates SASP of hepatic stellate cells (Lujambio et al., 2013). SASP also regulates genes affecting macrophage function. In fact, p53 signaling, through SASP, influences macrophage polarization (Lujambio et al., 2013). Precisely, induction of senescence by p53 activation in malignant hepatocytes showed a reduction in the tumor size caused by SASP‐mediated recruitment of immune cells to the tumors (Lujambio et al., 2013). Here, the results showed in vivo and in vitro that normal expression of p53 is required to ensure the homeostasis of OPN in liver and in HepG2 cells. Thus, p53‐OPN axis is required for maintaining the liver health during aging.

In summary, OPN deficiency increases the susceptibility of the liver to aging and aging‐associated liver disease. As OPN‐deficient mice become older, increased levels of senescence, ER stress, hepatosteatosis, DNA damage, fibrosis, and inflammation appear. The in vivo results are supported by in vitro findings. In addition, liver OPN is p53 regulated, and it plays a role in ensuring OPN secretion in response to senescence. The overall data suggest that OPN is a protective factor that counteracts senescence.

## 4 EXPERIMENTAL PROCEDURES

### 4.1 Human samples

This study included 123 patients; 42 were nonobese and 81 were obese. Individuals were considered nonobese when presenting a BMI lower than 30 kg/m^2^ and obese when the BMI was higher than 30 kg/m^2^. Liver biopsy was performed during laparoscopic cholecystectomy or during bariatric surgery. According to Kleiner criteria (Kleiner et al., 2005), of the obese group, 76 individuals had a clinical diagnosis of nonalcoholic fatty liver disease (NAFLD) and 5 were obese control individuals with normal liver (NL). The nonobese group was comprised of 13 individuals with NAFLD and 29 control individuals with NL. Steatosis was assessed as outlined by Kleiner et al. (2005). The study was performed in agreement with the Declaration of Helsinki and with local and national laws. The Human Ethics Committee of the University Hospital Santa Cristina and the Ethical committee of clinical research of the Basque Country approved the study procedures. Written informed consent was obtained from all patients before inclusion in the study.

### 4.2 Animals

3‐, 10‐, and 20‐month‐old WT and OPN‐KO female mice were used. Mice were maintained on a rodent chow diet (CD) (Teklad Global 18% Protein Rodent Diet 2018S; Harlan Laboratories INC., USA) or a rodent high‐fat diet (HFD) (Bioserv S3282, 60% Fat) and water ad libitum.

In addition, 3‐month‐old WT and p53‐KO mice were used to assess the role of p53 in OPN regulation. For assessing OPN expression in p53‐KO animals, mice were maintained on a rodent CD (Teklad Global 18% Protein Rodent Diet 2018S; Harlan Laboratories INC., USA) or a rodent HFD (Bioserv S3282, 60% Fat) and water ad libitum for 4 weeks. For assessing OPN expression after adenoviral over‐expression of p53, WT and p53‐KO mice were maintained in a rodent high‐fat diet (Research Diets D12,492; 60% fat, 5.24 kcal/g, Research Diets, New Brunswick, NJ) and water ad libitum for 11 weeks. Animal procedures were approved by the Ethics Committee for Animal Welfare of the University of the Basque Country UPV/EHU and were conducted in conformity with the EU Directives for animal experimentation.

### 4.3 In vivo adenoviral gene transfer

Wild‐type (WT) mice and p53‐KO mice were injected by tail vein injection with 100 ml of adenoviral vectors diluted in saline for over‐expression of hepatic p53. Adenoviral vectors activating p53 (SignaGen Laboratories, USA, ref # SL100,777) and GFP (SignaGen Laboratories, USA, ref # SL100,833) (1_109 VGml_1) were used.

### 4.4 Glucose and insulin tolerance tests

For glucose tolerance test (GTT), mice were fasted for 4 hr and then were administered a glucose solution (2 g/kg body weight) by oral gavage. Blood glucose levels were measured before glucose administration (time 0) and 15, 30, 60, and 120 min postgavage using blood strips. For insulin sensitivity test (ITT), mice were injected intraperitoneally 1 U insulin/kg body weight and glucose levels were measured as described above for glucose tolerance test.

### 4.5 Glucose and insulin measurement

Glucose blood levels were measured using blood strips. For insulin measurement, an ultrasensitive mouse Insulin ELISA kit (catalog #90080; Crystal Chem, Downers Grove, IL) was used following manufacturer's instructions.

### 4.6 *In vitro* studies

HepG2 and Hep3B cell lines were obtained from the American Type Culture Collection (ATCC). Cells were maintained in Dulbecco's modified Eagle's medium supplemented with fetal bovine serum 10% (v/v), penicillin‐streptomycin 1%, glutamine, and amphotericin B 1%. Cells were kept at 37°C within a 95% humidified atmosphere containing 5% carbon dioxide in an incubator. Depending on the experiment, cells were plated in 60‐mm culture dishes, 6‐well plates, or in 24‐well culture plates.

### 4.7 Cell transfection

For knocking‐down experiments, a commercial siRNA was used against osteopontin (SPP1 gene) and p53 (TP53 gene) (Ambion CA, USA). Negative controls were included in each assay by using Silencer™ Select Negative Control siRNA (Ambion CA, USA). Reverse transfection was performed using RNAiMAX lipofectamine (Invitrogen Life Technologies, USA) in Opti‐MEM media. Briefly, Lipofectamine and siRNA were diluted separately in Opti‐MEM and then dilutions were mixed and incubated for 20 min at RT. 24 hr after transfection, transfection media was removed and fresh treatment media was added. Gene silencing efficiency was confirmed by RNA expression analysis.

### 4.8 Palbociclib treatment

Senescence inductor Palbociclib 2 μM was dissolved in DMSO and filtered. 24 hr after transfection, HepG2 cells were exposed to palbociclib or vehicle, which was added to the regular complete media. Cells were collected 4 days after palbociclib treatment for their study.

### 4.9 Hydrogen peroxide treatment

Hydrogen peroxide (H_2_O_2_) was used for senescence induction. 24 hr after plating, cells were exposed to H_2_O_2_ 700 μM or vehicle for 1 hr. Then, treatment media was replaced and fresh media was added. Cells were collected 4 days after H_2_O_2_ treatment for their study.

### 4.10 Analysis of liver and serum lipid concentration

After homogenization of liver tissue or scraping cells, lipids were extracted as described before and detailed in Appendix S1.

### 4.11 Immunoassays

For the immunoblots, samples were separated to SDS‐PAGE and proteins were transferred to nitrocellulose membranes. Western blotting was performed using the primary antibody of interest.

### 4.12 [^3^H]Acetate and [^3^H]oleate incorporation

For *de novo* lipogenesis analysis, primary hepatocytes (1.5 × 10^6^ cells/plate) or liver slices (40 mg) were incubated with sodium [^3^H]acetate (84 mCi/ mmol;20 µCi/mL; 20 µM). For esterification analysis primary hepatocytes (1.5 × 10^6^ cells/plate) were incubated with [^3^H]oleate (54 mCi/mmol; 2 µCi/mL; 20 µM) and liver slices (40 mg) were incubated with [^3^H]oleate (54 mCi/mmol; 2 µCi/mL; 800 µM). At 240 min, cells and mediums were collected. To quantify the radioactivity incorporated from [^3^H]acetate and [^3^H]oleate into lipids, lipids were extracted and separated. After the scraping of the corresponding bands into a vial containing scintillation, liquid radioactivity was determined.

### 4.13 Fatty acid oxidation measurement

Beta oxidation was assessed as described before and detailed in Appendix S1.

### 4.14 Osteopontin ELISA

For quantification of serum and cell supernatant OPN concentration, a Human or Mouse OPN Quantikine ELISA kit (R&D Systems) was used following manufacturer's instructions.

### 4.15 Histochemistry

Paraffin‐embedded sections (5 μm thick) of formalin‐fixed liver samples or OCT‐embedded samples were used. All procedures are detailed in Appendix S1.

### 4.16 Total protein measurement

Protein concentration was measured using commercially Bicinchoninic Acid Reagent(Thermo Fisher Scientific Inc).

### 4.17 Statistical analysis

Data are represented as mean ± *SEM*. Differences between groups were tested using Student's *t* test, two‐way ANOVA. Significance was defined as *p* < 0.05. Correlation analysis was tested using the Pearson correlation test. These analyses were performed using GraphPad Prism software.

## CONFLICT OF INTEREST

MLM‐Ch is consultant of Mitotherapeutix.

## AUTHOR CONTRIBUTIONS

BG‐S and PA designed the study. BG‐S, DSDU, MN‐G, FG‐R, XB, IA, VGDJ, MJG‐R, LM, CM, and PA performed experiments and investigations. CG‐M, AG‐R, GE, PM, SG, MJG‐R, LC, and RN acquisition of samples and investigations. BG‐S, RN, MLM‐Ch, WS, and PA contributed to data analysis and discussion. BG‐S and PA wrote the paper, and all authors contributed to editing.

## Supporting information

Figure S1

Figure S2

Figure S3

Figure S4

Figure S5

Supplementary Material

## Data Availability

The data that support the findings of this study are available from the corresponding author upon reasonable request.
