# Supplementary figures and images for: Liver osteopontin is required to prevent the progression of age‐related nonalcoholic fatty liver disease
Source: Aging Cell. 2020 Jul 7;19(8):e13183. doi: 10.1111/acel.13183 (PMC7431823; doi:10.1111/acel.13183)

**A**

DNL 20m WT &amp; OPN-KO

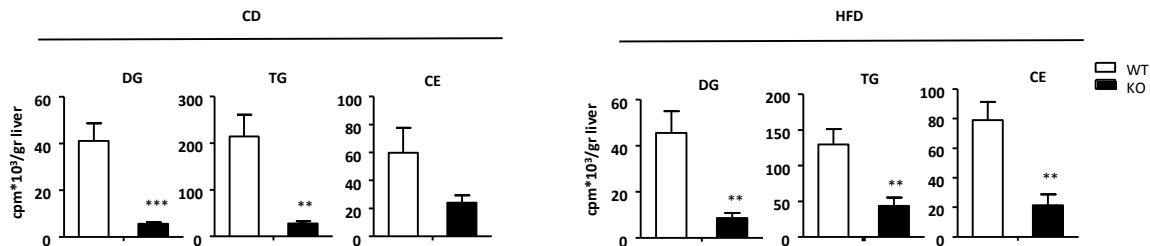**B**

Esterification of oleate 20m WT &amp; OPN-KO

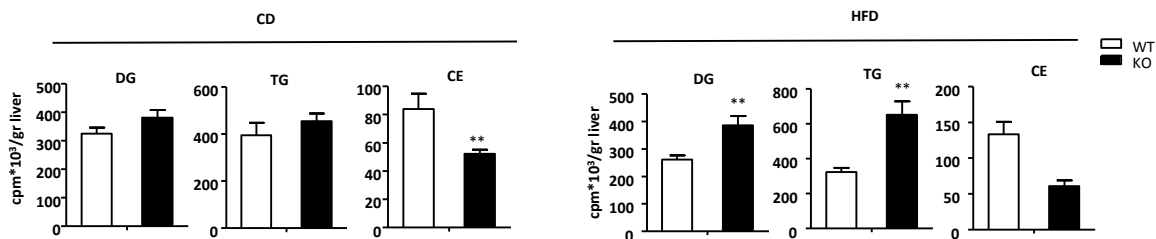**C**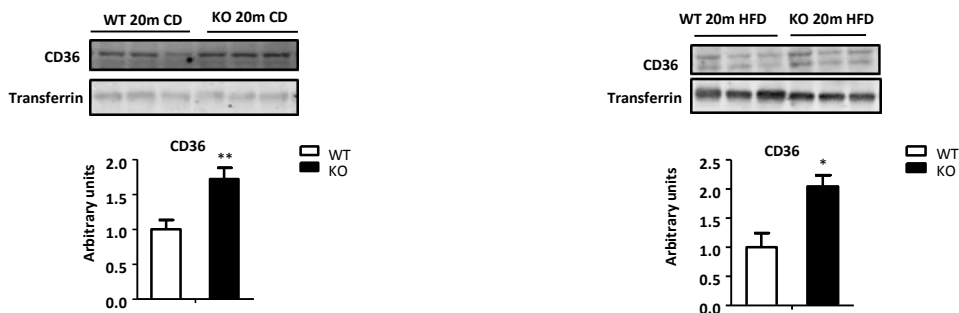**D**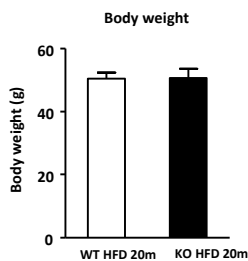**E**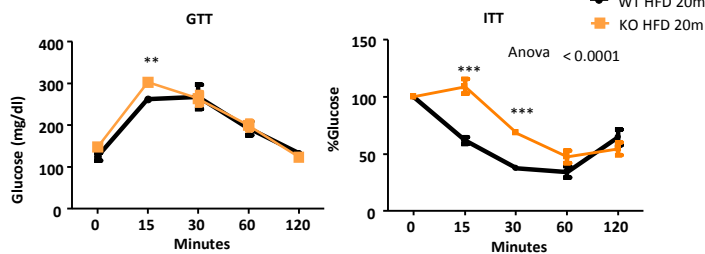**F**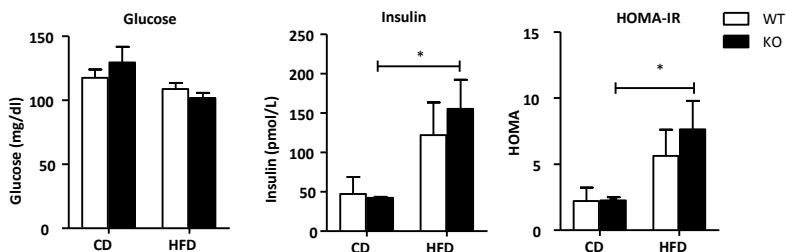

Supplement: Supplementary file 1 — Figure S1 [file ACEL-19-e13183-s001.pdf]

**A****Beta Oxidation**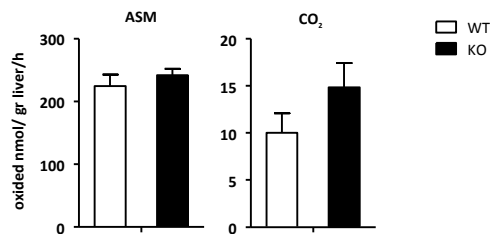**B**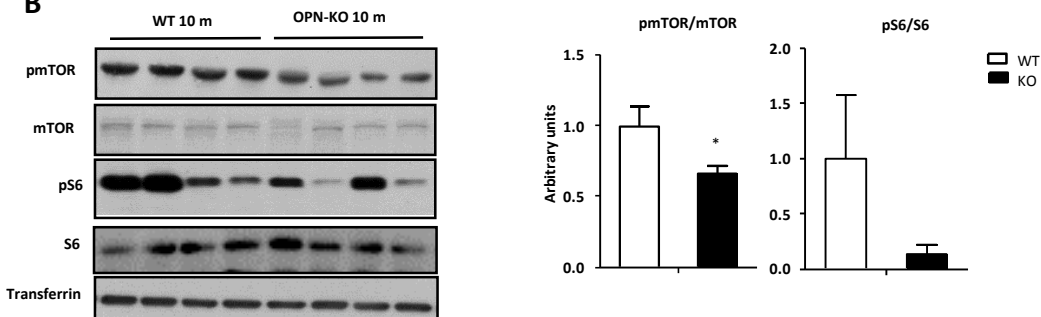**C**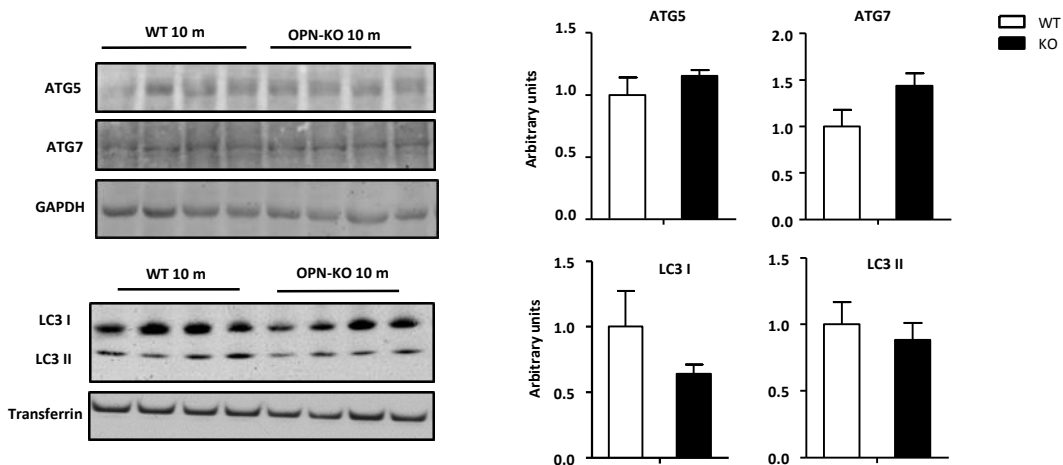**D**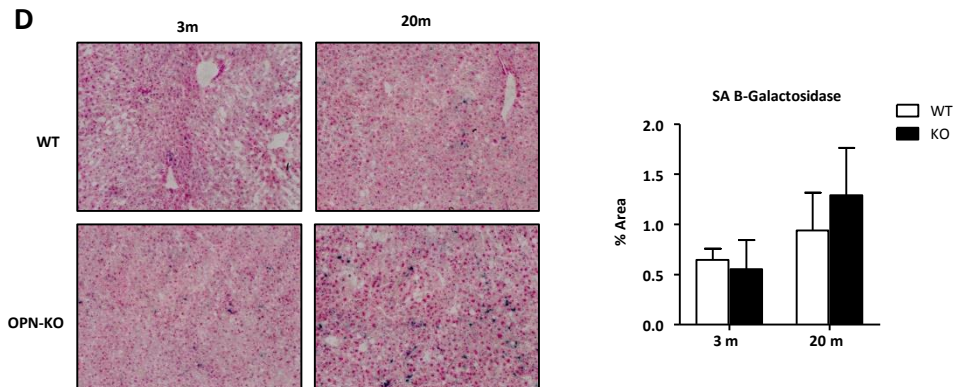

Supplement: Supplementary file 2 — Figure S2 [file ACEL-19-e13183-s002.pdf]

**A**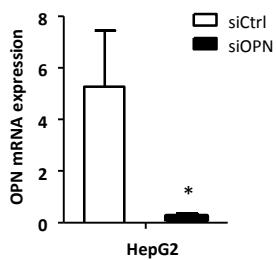**B**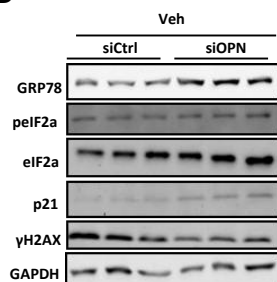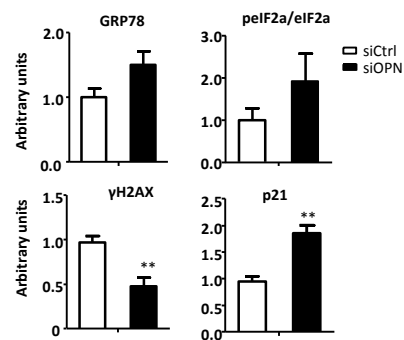**C**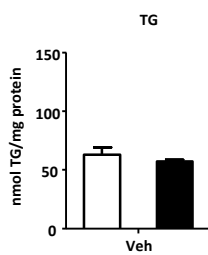**D**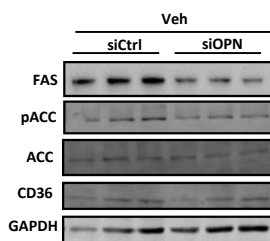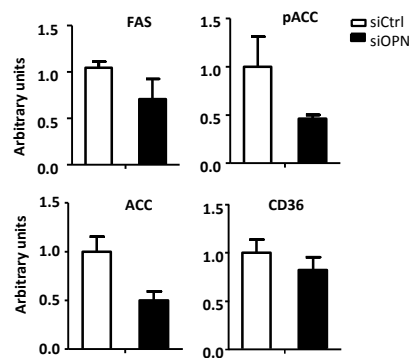

Supplement: Supplementary file 3 — Figure S3 [file ACEL-19-e13183-s003.pdf]

**A**

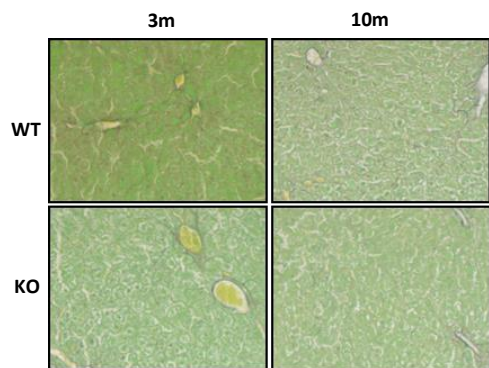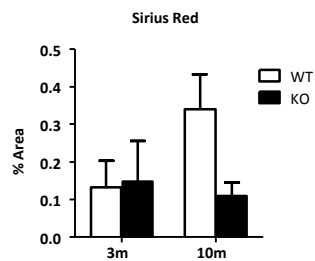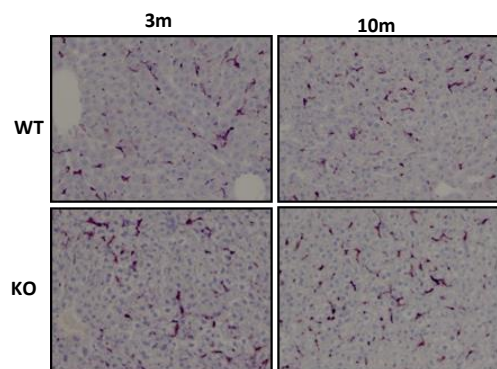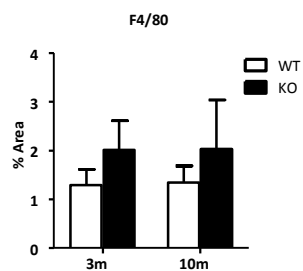

**B**

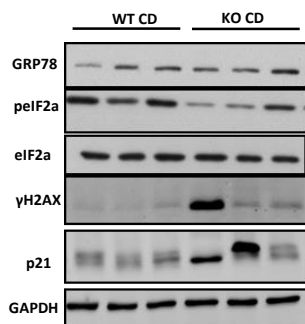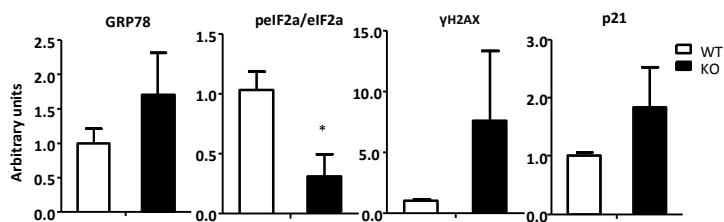

Supplement: Supplementary file 4 — Figure S4 [file ACEL-19-e13183-s004.pdf]

**A**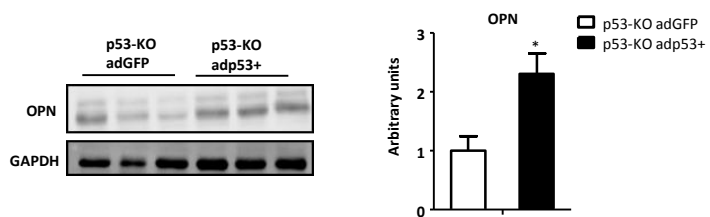**B**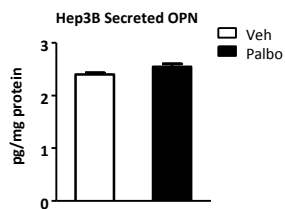**C**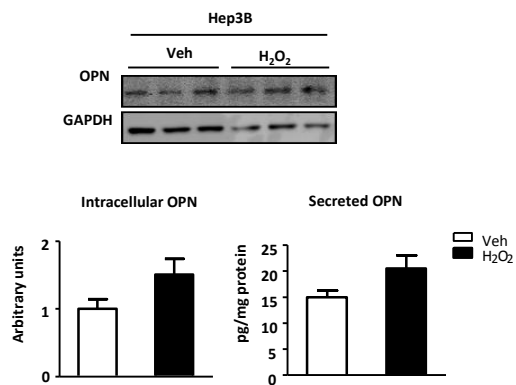

Supplement: Supplementary file 5 — Figure S5 [file ACEL-19-e13183-s005.pdf]
