# Supplementary material for: Liver osteopontin is required to prevent the progression of age‐related nonalcoholic fatty liver disease
Source: Aging Cell. 2020 Jul 7;19(8):e13183. doi: 10.1111/acel.13183 (PMC7431823; doi:10.1111/acel.13183)
Supplement: Supplementary file 6 — Supplementary Material [file ACEL-19-e13183-s006.doc]

**Liver osteopontin is required to prevent the progression of age-related nonalcoholic fatty-liver disease**

Beatriz Gómez-Santos^1^, Diego Saenz de Urturi^1^, Maitane Nuñez-García^1^, Francisco Gonzalez- Romero^1^, Xabier Buque^1,2^, Igor Aurrekoetxea^1,2^, Virginia Gutiérrez de Juan^3^, Maria J Gonzalez- Rellan^4^, Carmelo García-Monzón^5^, Águeda González-Rodríguez^5^, Lorena Mosterio^2^, Gaizka Errazti^2^, Patricia Mifsut^2^, Sonia Gaztambide^2^, Luis Castaño^2^, Cesar Martin^6^, Rubén Nogueiras^4^, María L Martinez-Chantar^3^, Wing-kin Syn1,7, 8, Patricia Aspichueta^1,2,*^

^1^Department of Physiology, Faculty of Medicine and Nursing, University of Basque Country UPV/EHU, Leioa, Spain; ^2^BioCruces Health Research Institute, Cruces University Hospital, Barakaldo, Spain; ^3^Center for Cooperative Research in Bioscience (CIC bioGUNE), CIBERehd, Bilbao, Spain; ^4^Department of Physiology, CIMUS, University of Santiago de Compostela- Instituto de Investigación Sanitaria, Santiago de Compostela, Spain; CIBER Fisiopatología de la Obesidad y Nutrición (CIBERobn), Spain; ^5^Liver Research Unit, Santa Cristina University Hospital, Instituto de Investigación Sanitaria Princesa, Madrid, Spain; Centro de Investigación Biomédica en Red de Enfermedades Hepáticas y Digestivas (CIBERehd), Spain; ^6^Biofisika Institute (UPV/EHU, CSIC) and Department of Biochemistry and Molecular Biology, UPV/EHU, Spain; ^7^Section of Gastroenterology, Ralph H Johnson, VAMC, Charleston. ^8^Division of Gastroenterology and Hepatology, Medical University of South Carolina, Charleston, USA

***Corresponding author**: Patricia Aspichueta, Department of Physiology, Faculty of Medicine and Nursing, University of the Basque Country UPV/EHU, Barrio Sarriena s/n, 48940 Leioa, Spain. Phone: +34 946012896; Fax: +34 946015662; e-mail: [patricia.aspichueta@ehu.eus](mailto:patricia.aspichueta@ehu.eus)

**Keywords**: Osteopontin, p53, lipid metabolism, nonalcoholic fatty liver disease, senescence, aging

Running title: Role of osteopontin in the aging liver

**Supporting Information list**

-Experimental Procedures

-References for experimental procedures

-Supplemental Figure Legends

**Experimental Procedures**

**Fatty acid oxidation measurement**

Beta-oxidation was assessed as described before (Huynh, Green, Koves, & Hirschey, 2014) and detailed in supporting information. Briefly, fresh liver pieces were homogenated in cold homogenization buffer and sonicated for 10 seconds. Then, the homogenates were centrifuged at 500 xg for 10 min at 4 °C. Approximately 500 µg of protein from the homogenates supernatant was used for the assay in a volume of 200 µL. The reaction started by adding 400 µL assay buffer containing ^14^C-palmitate palmitic acid (56 mCi/mmol; 0.4 μCi/mL; 500 μM) to the samples and was incubated for 1 h at 37 °C in eppendorf tubes with a whatman paper circle in the cap. The reaction was stopped by carefully adding 300 µL of perchloric acid 3 M and NaOH 1 M was added to impregnate the whatman cap.

After 2 h the Whatman caps were removed and the radioactivity associated was measured in a scillation counter. The eppendorf tubes were centrifuged at 21,000 xg 10 minutes at 4 °C. 400 µL from the supernatant were collected and the radioactivity was counted in a scintillation counter. The supernatant contained the acid soluble metabolites (ASM) and the whatman caps captured the released CO_2_.

**Histochemistry**

Paraffin-embedded sections (5 μm thick) of formalin-fixed liver samples or OCT embedded samples were used.

For *Hematoxylin and eosin,* sections were submerged for 5 min in Harris hematoxylin, and then sections were washed in water for 5 min and stained in eosin for 15 min.

For *Sirius Red* staining, sections were stained with Sirius red solution (0.01% Fast Green FCF/0.1% Sirius red in picric acid, Sigma Aldrich) for 30 min.

For *Masson Trichrome*, samples were stained in Weigert's iron hematoxylin working solution for 10 min. After washing were stained in Biebrich scarlet-acid fuchsin solution for 10 min. Samples were differentiated in phosphomolybdic-phosphotungstic acid solution for 10 min. Then, were transferred to aniline blue solution and stained for 5 min, rinsed briefly in distilled water and differentiated in 1% acetic acid solution for 2-5 min. Finally, samples were dehydrated and mounted. An expert histopathologist from Veterinary Faculty, University Complutense of Madrid, performed tissue staining and evaluation.

For *SA-β Galactosidase* staining 10 μm thick frozen tissue sections were fixed with 4% NBF/0.5 % Glutaraldehyde solution. For the staining solution, a citric acid 40 mM was prepared in sodium phosphate heptahydrate 0.2 M pH 6. Potassium ferricyanide and potassium ferrocyanide was added to this solution, final concentration 5mM, MgCl2 2 mM and NaCl 150 mM. Staining solution was heated at 37 °C and 1 mg/mL X-Gal was added. Samples were incubated overnight in a humid chamber. Finally, samples were counterstained with 1% Fast Red for 2 min.

For in-vitro *SA-β Galactosidase* staining: after treatments, cells were washed in PBS and fixed in formaldehyde 3.7%. After this, staining solution (described above) was added to the wells and incubated overnight at 37 ºC. 4 fields per well were counted for total and positive cells.

For *F4/80 staining,* sections were unmasked and subjected to peroxide blocking, 3% H_2_O_2_ in PBS, 10 min RT. Samples were blocked with goat anti-mouse FAB fragment (Jackson Immunoresearch, USA) (1:10, 1h RT) and then blocked with 5% goat serum (30 min, RT). Then, sections were incubated in a humid chamber with the primary antibody followed by Envision anti-mouse (DAKO) HRP conjugated secondary antibody incubation (30 min, RT). Colorimetric detections were confirmed with vector VIP chromogen (Vector) and sections were counterstained with hematoxylin.

For *p53* inmunohistochemistry 10 μm thick frozen tissue sections were fixed with 10% NBF and included in paraffin. Endogenous peroxidase was blocked with 3% H_2_O_2_ for 10 min, blocked for 30 min with 5% serum in PBS 1h at RT with a 1:10 FAB fraction. Samples were washed with PBS and subsequently incubated 1h at 37ºC with p53 antibody (sc-126) at 1:50 dilution. Samples were washed with PBS and incubated for half an hour with Immpress anti-mouse (MP-7452). Finally, they were contrasted with Mayer's hematoxylin for 1 minute, washed with running water, dehydrated and mounted with DPX. For the analysis 5 photos were taken at a magnification of 40x and analyzed with the Cell profiler.

**Analysis of liver and serum lipid concentration**

After homogenization of liver tissue or scraping cells, lipids were extracted as described before and detailed in supporting information. (FOLCH, LEES, & SLOANE STANLEY, 1957). TG, CE and DG were quantified as described previously (Ruiz & Ochoa, 1997) and TGs, DGs and CEs were quantified. Free fatty acids (FA) were quantified using a commercially available kit (Wako, Germany). Serum TG, Chol and FAs were quantified using commercially available kits (A. Menarini Diagnostics, Italy; Wako, Germany).

**References**

FOLCH, J., LEES, M., & SLOANE STANLEY, G. H. (1957). A simple method for the isolation and purification of total lipides from animal tissues. *The Journal of Biological Chemistry, 226*(1), 497-509.

Huynh, F. K., Green, M. F., Koves, T. R., & Hirschey, M. D. (2014). Measurement of fatty acid oxidation rates in animal tissues and cell lines. *Methods in enzymology* (pp. 391-405) Elsevier.

Ruiz, J. I., & Ochoa, B. (1997). Quantification in the subnanomolar range of phospholipids and neutral lipids by monodimensional thin-layer chromatography and image analysis. *Journal of Lipid Research, 38*(7), 1482-1489.

**Supplementary Figures**

**Supplementary 1. Fatty acid uptake and esterification increases in 20m OPN-KO mice liver.** (A) *De novo* lipogenesis was evaluated by [^3^H]acetate incorporation into diglycerides (DG), triglycerides (TG) and cholesteryls esters (CE) was evaluated in 20m CD and HFD WT and OPN-KO mice. (B) Esterification was evaluated by [^3^H]oleate incorporation into DG, TG and CE in 20m CD and HFD WT and KO mice. (D) CD36 protein levels of 20m CD and HFD WT and OPN-KO mice were assessed by immunoblotting using transferrin as loading control (n=4-5). (D) Body weight was measured in 20 month-old (m) WT and OPN-KO (KO) high fat diet (HFD) fed mice. (E) Glucose (GTT) and insulin tolerance tests (ITT) were performed in HFD fed 20m WT and OPN-KO mice. (F) Glucose, insulin fed levels and HOMA-IR index were measured and calculated in 20m WT and OPN-KO CD and HFD fed mice. Values are means ±SEM of n=4-6. Significant differences are denoted by ANOVA and Student’s t test.

**Supplementary 2. mTOR signaling was altered while autophagy markers maintained unchanged in 10m OPN-KO mice liver.** (A) Fatty acid oxidation rate was measured in liver homogenates of 10m WT and OPN-KO mice. Values are means ±SEM of n=7-8. (B) Phosphorylated mTOR (pmTOR), total mTOR, phosphorylated S6 (pS6) and total S6 protein levels of 10m WT and OPN-KO mice were assessed by immunoblotting using transferrin as loading control (n=5-6). (C) ATG5, ATG7 LC3B protein levels where evaluated using glyceraldehyde -3-phosphate dehydrogenase (GAPDH) or transferrin as a loading control (n=5-6). (D) Liver senescence-associated (SA) β-Galactosidase positive area percentage was quantified in 3 and 20m WT and OPN-KO mice (n=4-5). Values are means ±SEM. Significant differences are denoted by *p<0.05, **p<0.01 and ***p<0.001 (Student’s t test).

**Supplementary 3. TG content and ER stress markers are unaltered in siOPN cells.** (A) mRNA expression of SPP1 gene was measured in HepG2 cells treated with a Ctrl siRNA and SPP1 siRNA. Values are means ±SEM (n=3). (B) In HepG2 cells GRP78, total and phosphorylated eIF2α, p21 and γH2AX protein levels were measured by immunoblotting using glyceraldehyde -3-phosphate dehydrogenase (GAPDH) as loading control in siCtrl and siOPN HepG2 cells treated with Veh (n=4-6). (C) Intracellular TG levels were measured in siCtrl and siOPN HepG2 cells treated with Veh (D) together with FAS, total and phosphorylated acetyl-CoA carboxylase (ACC) and CD36 protein levels using glyceraldehyde -3-phosphate dehydrogenase (GAPDH) as a loading control (n=4). Values are means ±SEM. Significant differences are denoted by *p<0.05, **p<0.01 and ***p<0.001 (Student’s t test).

**Supplementary 4. Fibrosis and inflammation markers are unaltered in 10m OPN-KO mice.** (A) Sirius Red and F4/80 stainings were performed to assess tissue fibrosis and inflammation in 3 and 10 month-old (m) WT and OPN-KO mice (n=4-6). (B) Immunoblot analysis of GRP78, total and phosphorylated eIF2a, γH2Ax and p21 protein levels from liver extract were assessed using glyceraldehyde -3-phosphate dehydrogenase (GAPDH) as a loading control in 20 m mice fed a chow diet (CD) (n=4-5). Values are means ±SEM. Significant differences are denoted by *p<0.05, **p<0.01 and ***p<0.001 when comparing WT and OPN-KO mice (Student’s t test).

**Supplementary 5. OPN cellular content and secretion maintains unaltered in Hep3B cells when senescence is induced.** (A) Liver OPN protein levels were evaluated in p53-KO mice fed a HFD injected with p53 dominant positive adenovirus (adp53) and GFP (adGFP), using GAPDH as a loading control (n=4-6). (B) OPN protein levels from Hep3B cells were measured by immunoblotting using glyceraldehyde -3-phosphate dehydrogenase (GAPDH) as loading control after treatment with hydrogen peroxide (H_2_O_2_). Secreted OPN in the media was measured by ELISA (n=4-6). (C) Secreted OPN levels were measured by ELISA in Hep3B cells treated with palbocilcib (Palbo) (n=4-6). Values are means ±SEM. Significant differences are denoted by *p<0.05, **p<0.01 and ***p<0.001 (Student’s t test).
